# Association among peripatellar fat pad edema and related patellofemoral maltracking parameters: a case-control magnetic resonance imaging study

**DOI:** 10.1186/s12891-023-06827-7

**Published:** 2023-08-25

**Authors:** Liangjing Lyu, Yongliang Li, Jingyu Zhong, Weiwu Yao

**Affiliations:** Department of Radiology, Tongren Hospital, Shanghai Jiao Tong University School of Medicine, No. 1111 Xianxia Road, Shanghai, 200336 China

**Keywords:** Fat pad, Edema, MRI

## Abstract

**Background:**

The peripatellar fat pads are critical for protective cushioning during movement, and their endocrine function has been shown to affect osteoarthritis. Magnetic resonance imaging (MRI) is frequently used to visualize edema of the peripatellar fat pads due to injury. In this study, we aimed to assess the relationship between peripatellar fat pad edema and patellofemoral maltracking MRI parameters and investigate the association among cases of peripatellar fat pad edema.

**Methods:**

Age- and sex-matched peripatellar fat pad edema cases were identified and divided into superolateral Hoffa, quadriceps, and prefemoral groups. Images were assessed according to tibial tuberosity lateralization, trochlear dysplasia, patellar alta, patellar tilt, and bisect offset. McNemar’s test or paired t-tests and Spearman’s correlation were used for statistical analysis. Interobserver agreement was assessed with the intraclass correlation coefficient.

**Results:**

Of 1210 MRI scans, 50, 68, and 42 cases were in the superolateral Hoffa, quadriceps, and prefemoral groups, respectively. Subjects with superolateral Hoffa fat pad edema had a lower lateral trochlear inclination (p = 0.028), higher Insall-Salvati (p < 0.001) and modified Insall-Salvati (p = 0.021) ratios, and lower patellotrochlear index (p < 0.001) than controls. The prefemoral group had a lower lateral trochlear inclination (p = 0.014) and higher Insall-Salvati (p < 0.001) and modified Insall-Salvati (p = 0.004) ratios compared with the control group. In contrast, the patellotrochlear index (p = 0.001) was lower. Mean patellar tilt angle (p = 0.019) and mean bisect offset (p = 0.005) were significantly different between cases and controls. The quadriceps group showed no association. Superolateral Hoffa was positively correlated with prefemoral (p < 0.001, r = 0.408) and negatively correlated with quadriceps (p < 0.001, r = -0.500) fat pad edema.

**Conclusions:**

Superolateral Hoffa and prefemoral fat pad edemas were associated with patellar maltracking parameters. Quadriceps fat pad edema and maltracking parameters were not associated. Superolateral Hoffa fat pad edema was positively correlated with prefemoral and negatively correlated with quadriceps fat pad edema.

## Background

Peripatellar fat pads are intracapsular extrasynovial adipose tissues that change shape, size, position, and pressure during movement to act as flexible, elastic, and displaceable protective cushions [1]. The peripatellar fat pad has gained academic attention due to its importance in biomechanics [2] and endocrine effects on osteoarthritis [3, 4].

There are three types of peripatellar fat pads: quadriceps (QFP), prefemoral (PFP), and infrapatellar (IFP, or Hoffa fat pad). During normal knee flexion-extension, the QFP interposes between the quadriceps tendon anteriorly and the femoral condyle posteriorly, while the PFP is anterior to the femur and is separated anteriorly from the quadriceps fat pad by the suprapatellar bursa. The IFP is located between the patellar tendon, femoral condyles, and the tibial plateau. Sustained friction and repetitive microtrauma can lead to peripatellar fat pad impingement, reported as chronic anterior knee pain and fat pad edema observed on magnetic resonance imaging (MRI). The IFP is organized into lobules defined by thin connective septa [5, 6]: large lobules with superficial septa in the superficial part near the patellar tendon and small lobules with thick septa in the deep part. IFP impingement refers to superolateral Hoffa fat pad edema (SHFPE) near the patellar tendon but not in the deep section, and edema of the deep section is associated with osteoarthritis, which is beyond the scope of our discussion. Therefore, in this study, we use the term “SHFPE” instead of “IFP edema.”

SHFPE has a high reported prevalence of 13% in middle-aged individuals [7]. QFP edema (QFPE) is also seen in 12–14% of patients undergoing knee MRI [8]. However, the prevalence of PFP edema (PFPE) remains unknown owing to its rarity. On the other hand, Patellofemoral maltracking is a dynamic abnormality in the engagement of the patella and femur [1]. This pathologic condition, which contributes to changing patellofemoral load and elevating joint stress, may finally cause patellofemoral pain, which presents as pain around the patella and is exacerbated by squatting, going up or down stairs, and prolonged sitting. Patellofemoral maltracking was thought to be potentially modifiable by taping or bracing technology for symptom improvement. Therefore, it gained attention in the field of sports medicine. [9]. Several parameters of patellofemoral maltracking were related to SHFPE in patients with knee pain [10, 11], indicating impingement [9]. Recent studies [7, 12] have focused on SHFPE and confirmed the relationship between SHFPE and patellofemoral maltracking. However, few studies [13, 14] have been conducted on QFPE, and no studies focus on PFPE. Furthermore, the related morphologic knee features, clinical symptoms, and management of the three kinds of peripatellar fat pad edema appear to be different [1, 8, 10, 13].

We aimed to assess the relationship between three kinds of peripatellar fat pad edema and patellofemoral maltracking MRI parameters and investigate the potential association among these types of peripatellar fat pad edema.

## Methods

Ethical approval was obtained for this retrospective analysis from the Institutional Review Board of Shanghai Tongren Hospital (approval number: 2022-044-01). Written informed consent was waived by Institutional Review Board of Shanghai Tongren Hospital Ethic committee.

### Study population

**Fig. 1:**
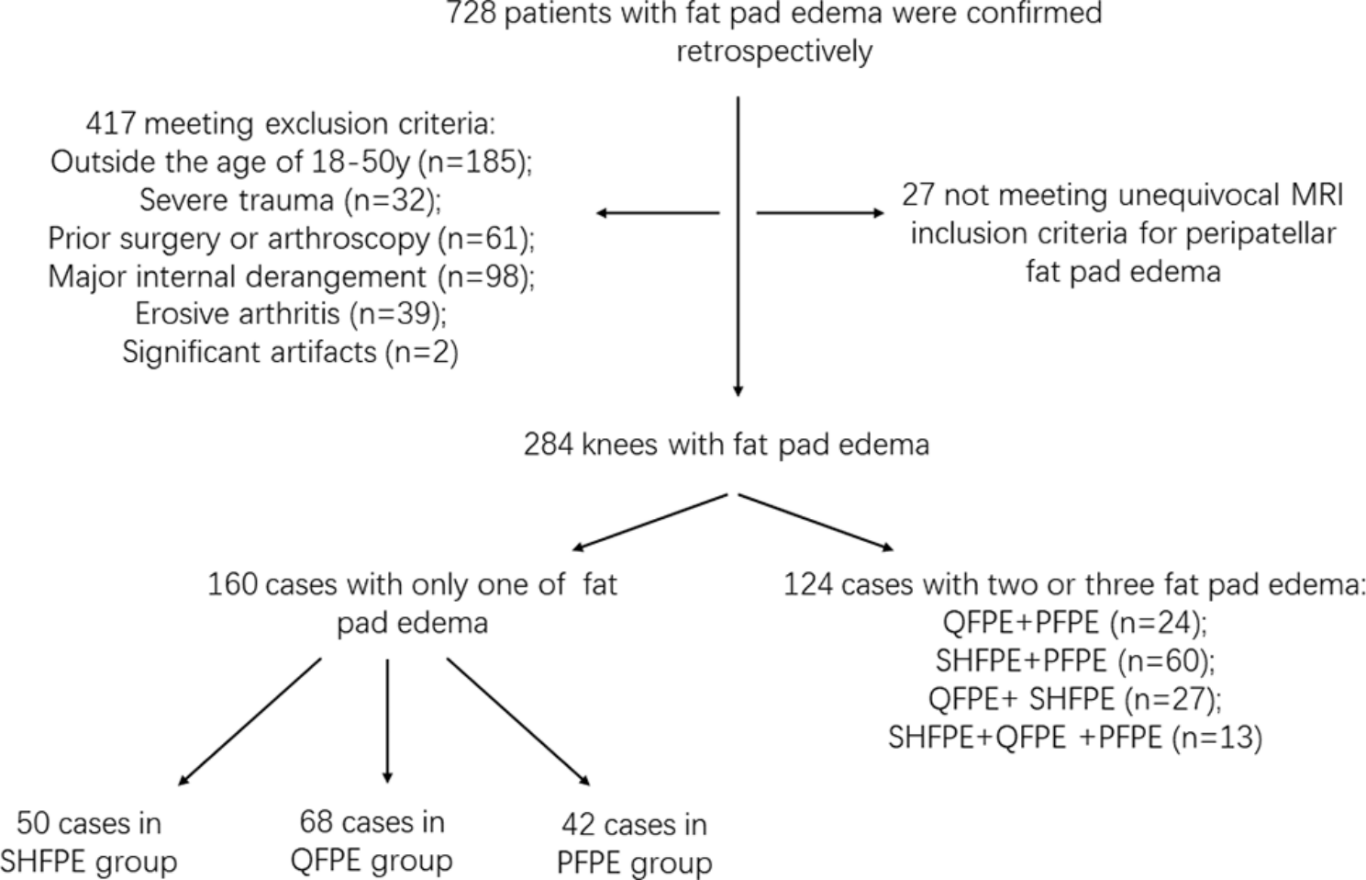
Flowchart summarizing the selection process of peripatellar fat pad edema cases. MRI, magnetic resonance imaging; QFPE, quadriceps fat pad edema; PFPE, prefemoral fat pad edema; SHFPE, superolateral Hoffa fat pad edema

To assess the relationship between three kinds of peripatellar fat pad edema and patellofemoral maltracking MRI parameters and investigate the potential association among these types of peripatellar fat pad edema, we reviewed knee MRI data from 1210 consecutive patients in our radiology database during the first half of 2021 to identify the presence and location of cases with peripatellar fat pad edema. Peripatellar fat pad edema was defined on MRI as a focal increased signal on proton density weight (PDW) sequences and decreased signal on T1-weighted (T1W) sequences. Sequences were compared with a normal fat pad. For IPF, only SHFPE was included in this study rather than the deep part. Patients were included based on a consensus reviewed by a board-certified radiologist with 7 years of musculoskeletal experience and one with 27 years of experience. The exclusion criteria were patients outside the ages of 18–50 years, severe knee trauma history (such as dislocation or fracture), prior surgery or arthroscopy, major internal derangement (such as a torn meniscus, tendon, or cruciate ligament), and evidence of erosive arthritis. MRI with significant artifacts was excluded. Subjects with only one instance of fat pad edema were divided into SHFPE, QFPE, and PFPE groups. For each group, age- and sex-matched control subjects with no fat pad edema on MRI were allotted randomly from our database during the first half of 2021, with a case:control ratio of 1:1. The control subjects were subjected to the same exclusion criteria (Fig. 1).

### MRI technique

All MRI scans were performed using a 3.0-T MR scanner (MAGNETOM Skyra; Siemens Healthineers, Erlangen, Germany). The imaging protocol included sagittal T1W sequences with 550–650/15 ms repetition time/echo time and fat-saturated coronal, sagittal, and axial PDW sequences with 2500–4000/30 ms repetition time/echo time. For all sequences, a 15-cm field of view and 3-mm slice thickness were used and subsequently performed using 15-channel dedicated phased-array knee coils. Patients were positioned with mild flexion (15°-20°) and fixed with a cotton cushion.

### Patellofemoral maltracking MRI parameter assessment

We carefully selected several patellofemoral maltracking parameters after reviewing previous studies [7, 11, 15–17], including lateralization of the tibial tubercle (TT-TG [tibial tuberosity-trochlear groove]) distance and TT-TG index, trochlear morphology (trochlear depth, lateral trochlear inclination angle), and patellar parameters (patella alta, patellar tilt angle, and bisect offset). Two board-certified radiologists measured these parameters (27 and 7 years of experience in musculoskeletal MRI, respectively) in both case and control groups. The averages of both readers’ measurements were used.

**Fig. 2:**
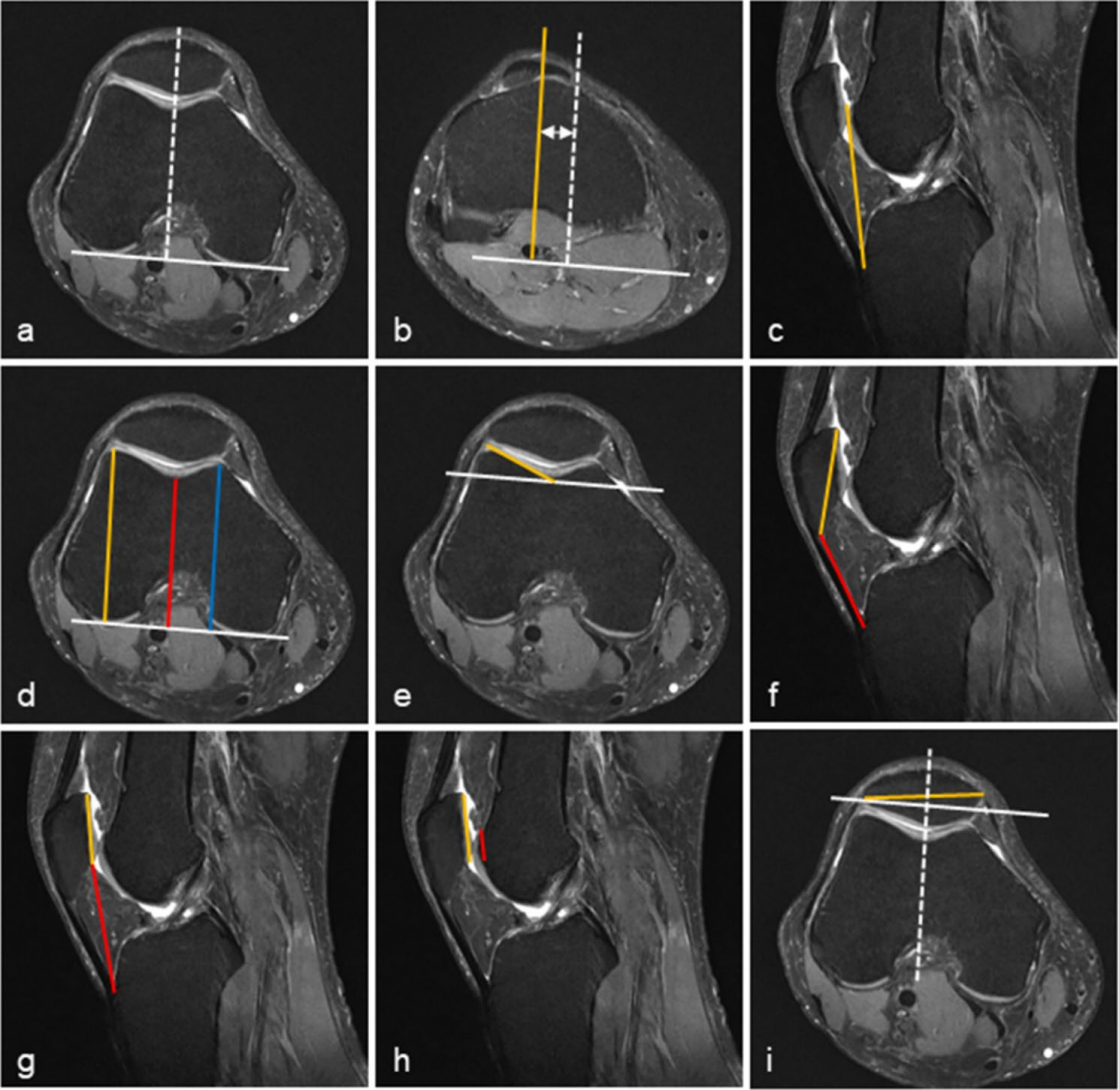
Measurements of the patellofemoral maltracking magnetic resonance imaging parameters. **a** A baseline (white line) was drawn on the slice where the femoral condyles were at the most posterior. A white dashed line was drawn perpendicular to the baseline, passing through the trochlear groove (TG). **b** Both lines were transferred inferiorly to the level of patellar tendon attachment. Another perpendicular line (yellow line) was drawn, passing through the center of the patellar tendon attachment (TT). The double arrow shows the TT-TG distance. **c** The distance (yellow line) between the TG entrance (TE) and patellar tendon attachment (TT) was TT-TE. TT-TG index=(TT–TG)/(TT–TE). **d** Three lines were drawn perpendicular to the baseline: one from the deepest point of the TG (red line) to the baseline (white line), the others from the medial (blue line) and lateral (yellow line) condyles’ anterior endpoints to the baseline. Trochlear depth was measured by subtracting perpendicular distances of the TG (red line) from the average (blue line and yellow line). **e** Lateral trochlear inclination was the angle between the baseline (white line) and the line parallel to the lateral trochlear facet subchondral bone (yellow line). **f** The Insall-Salvati ratio (ISR) was the ratio between patellar tendon length (red line) and patellar height (yellow line). **g** The modified ISR was the ratio between the distance (red line) from the distal end of the patellar cartilage to the TT and the length (yellow line) of the patellar cartilage. **h** The patellotrochlear index (PTI) was the ratio between the vertical patellar cartilage distance (yellow line) and the distance (red line) of trochlear cartilage in contact with the patella. **i** Patellar tilt angle was the angle between the patellar width line (yellow line) and baseline (white line). Bisect offset was defined as the percentage of patella width (yellow line) lateral to midline (white dashed line)

The TT-TG distance, TT-TG index, trochlear depth, and lateral trochlear inclination were measured on axial PDW sequences. A baseline (Fig. 2a) was drawn by connecting the medial and lateral posterior femoral condyles in the axial PDW section with the medial and lateral posterior femoral condyles at the most posterior. Two parallel lines were drawn perpendicular to the baseline, one passing through the deepest trochlear groove and one through the center of the patellar tendon attachment. The distance between the two lines represents the TT-TG distance (Fig. 2b) [18, 19]. The TT-TG index [20] is the ratio of the TT-TG distance and the distance (Fig. 2c) from the trochlear groove entrance to the patellar tendon attachment on the sagittal PDW image.

To evaluate trochlear depth [15], three lines were drawn perpendicular to the baseline: (a) maximal anteroposterior osseous distance of the medial femoral condyle, (b) maximal anteroposterior osseous distance of the lateral femoral condyle, and (c) distance from the deepest trochlear groove to the baseline. [(a + b)/2] − c is the trochlear depth (Fig. 2d). The lateral trochlear inclination angle [21] was the angle formed by the lateral facet line and the baseline (Fig. 2e).

The Insall-Salvati ratio (ISR), modified ISR (MISR), and patellotrochlear index (PTI) were measured using sagittal PDW sequences. The ISR [22, 23] was measured as the ratio of the patellar tendon’s length to the patellar length (Fig. 2f). The MISR [16] was measured as the ratio between the distance from the distal end of the patellar cartilage to the tibial tuberosity and the length of the patellar cartilage (Fig. 2g). The PTI [16] was measured as the ratio between the vertical patellar cartilage distance and the distance of trochlear cartilage contacted with the patella (Fig. 2h). The patellar tilt angle [17] was defined as the angle between the baseline and the maximal patellar width (Fig. 2i). Bisect offset [24] was defined as the percentage of the patella lateral to the midline (Fig. 2i).

### Peripatellar fat pad edema assessment

Peripatellar fat pad edema was defined as unambiguous hyperintensity on fat-suppressed PDW sequences (axial and sagittal) and hypointensity on T1W sequences. The two abovementioned readers evaluated each image and graded each case using the following scales based on a consensus.

**Fig. 3:**
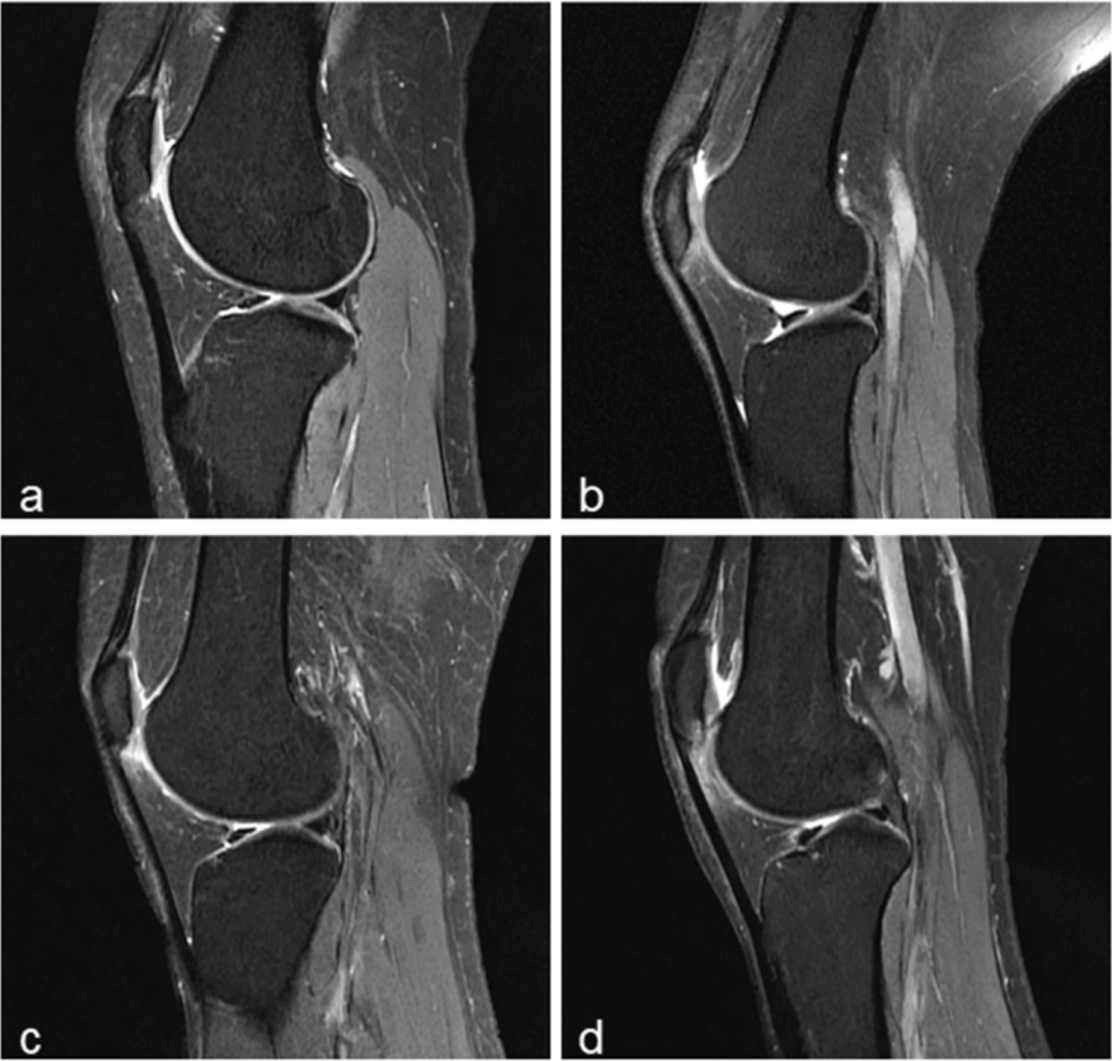
Superolateral Hoffa fat pad edema (SHFPE). Sagittal magnetic resonance imaging of the knee shows the SHFPE scoring system for **a** normal, **b** mild or grade 1, **c** moderate or grade 2, and **d** severe or grade 3 SHFPE.

SHFPE (Fig. 3) was evaluated using a 4-point scale [25] based on edema size on sagittal PDW sequences: grade 0, absent; grade 1, mild (10% or less of patellar tendon length); grade 2, moderate (10–30% of patellar tendon length); grade 3, severe (more than 30% of patellar tendon length).

**Fig. 4:**
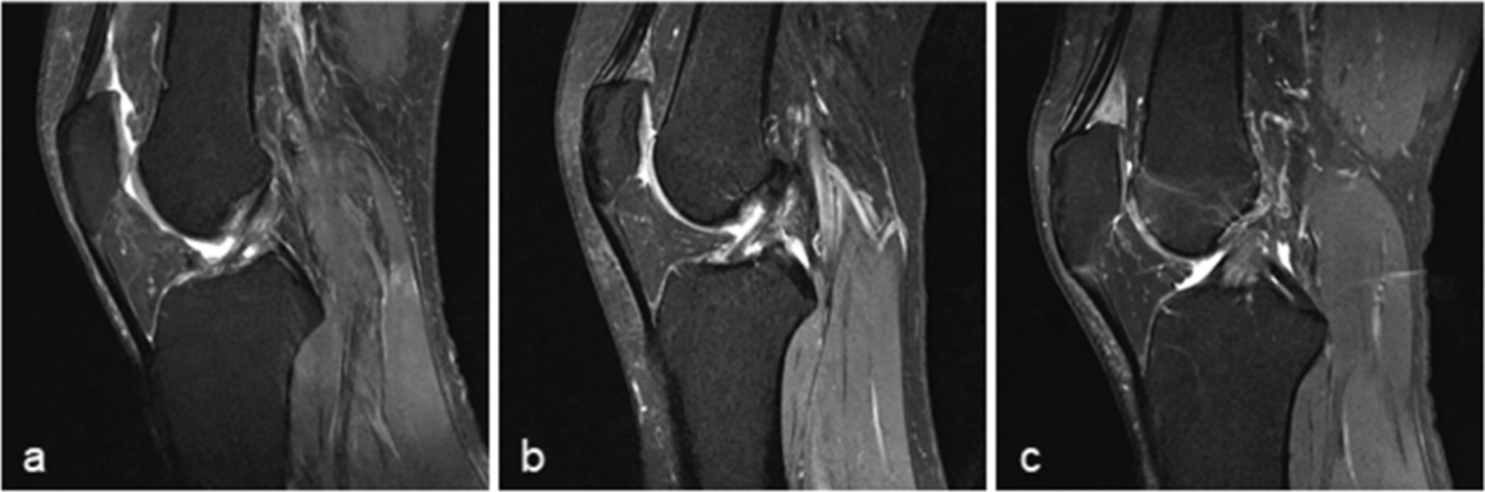
Suprapatellar quadriceps fat pad edema (QFPE). Sagittal magnetic resonance imaging of the knee shows the QFPE scoring system for **a** normal; **b** mild or intermediate, grade 1; and **c** severe or grade 2 QFPE.

**Fig. 5:**
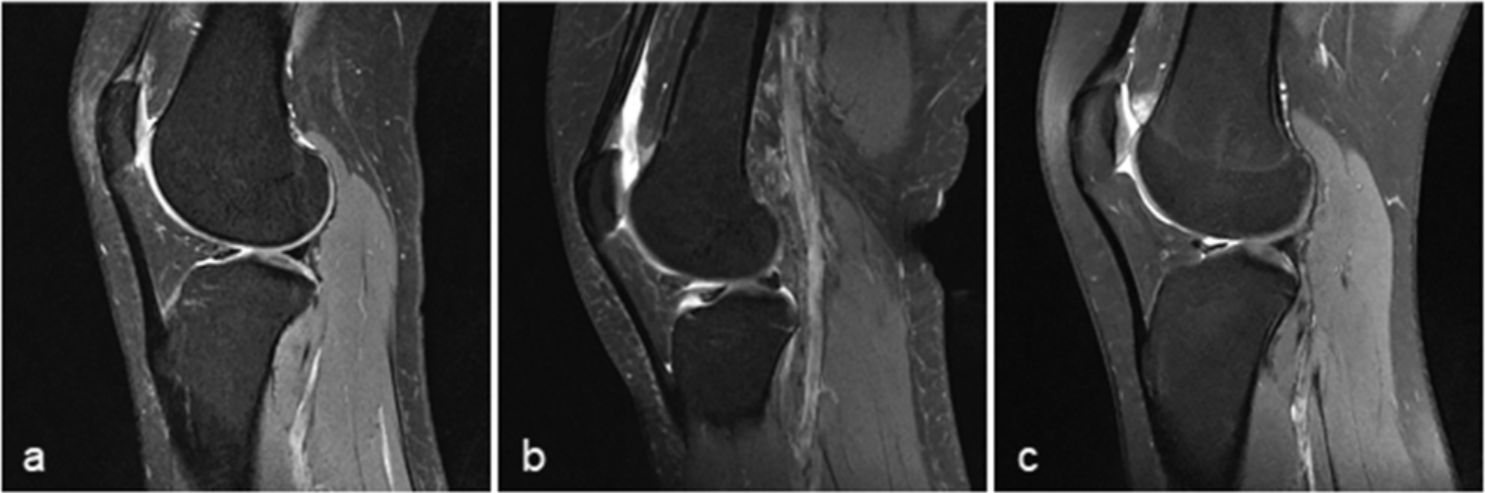
Prefemoral fat pad edema (PFPE). Sagittal magnetic resonance imaging of the knee shows the PFPE scoring system for **a** normal; **b** mild or intermediate, grade 1; and **c** severe or grade 2 PFPE.

QFPE (Fig. 4) and PFPE (Fig. 5) were determined visually from sagittal PDW sequences using a 3-point scale on the signal intensity of edema, other normal fat pads, and gastrocnemius muscle as a reference: grade 0, absent; grade 1, mild or intermediate (signal intensity higher than that of normal fat pad but not higher than that of gastrocnemius muscle); grade 2, severe (signal intensity higher than that of gastrocnemius muscle). We partly used the grading system suggested by Erber et al. [14] for reference, but we took their grade A (mild) and grade B (intermediate) together as grade 1 to avoid any ambiguity.

### Statistical analysis

We used McNemar’s test or paired t-test to compare the clinical characteristics and patellofemoral maltracking parameters between the case and control groups. After grading the fat pad edema groups, we used Spearman correlation analysis to study the association between SHFPE and other fat pad edemas. The intraclass correlation coefficient was used for inter- and intra-observer reliability analysis as follows: poor, intraclass correlation coefficient < 0.50; moderate, intraclass correlation coefficient = 0.50–0.75; good, intraclass correlation coefficient = 0.75–0.90; and excellent, intraclass correlation coefficient > 0.90 [26]. Statistical analysis was performed using SPSS (version 23; IBM, Armonk, NY), with significance set at p = 0.05.

## Results

Of 1210 knee MRI scans, 728 showed fat pad edema. After reviewing the scans and applying the exclusion criteria, the SHFPE, QFPE, and PFPE groups included 50, 68, and 42 case-control pairs, respectively. In addition, there were 124 cases with two or three different fat pad edemas. The clinical background of the subjects is summarized in Table 1. The SHFPE group included 34 females (68%), with a mean age of 36.4 ± 1.5 years. The QFPE group included 32 females (47%), with a mean age of 35.8 ± 6.8 years. The PFPE group included 29 females (69%), with a mean age of 36.4 ± 7.7 years. The SHFPE group had a higher prevalence of patellar tenderness (inferior) than the control group (38 vs. 20; p = 0.001). The PFPE group had a higher prevalence of patellar tenderness (superior) than the control group (27 vs. 14; p = 0.019). The other clinical characteristics were not significantly different between cases and controls. In the interobserver reliability evaluation (Table 2), there was excellent reliability for TT-TG, ISR, and MISR (0.944, 0.955, and 0.966, respectively); good reliability for TT-TG index, PTI, and bisect offset (0.796, 0.885, and 0.859, respectively); and moderate reliability for trochlear depth, lateral trochlear inclination, and patellar tilt angle (0.520, 0.559, and 0.617, respectively). In the intra-observer reliability evaluation (Table 2), there was excellent reliability for TT-TG, ISR, bisect offset, and MISR (0.959, 0.978, 0.913, and 0.983, respectively); good reliability for TT-TG index and PTI (0.885 and 0.893, respectively); and moderate reliability for trochlear depth, lateral trochlear inclination, and patellar tilt angle (0.667, 0.608, and 0.647, respectively).

**Table 1:** Clinical background of knee magnetic resonance imaging in the included subjects

Clinical information	SHFPE group cases	SHFPE group controls	p^a^	QFPE group cases	QFPE group controls	p^b^	PFPE group cases	PFPE group controls	p^c^	Mixed group cases^d^
	(n = 50)	(n = 50)		(n = 68)	(n = 68)		(n = 42)	(n = 42)		(n = 116)
Age^*^ (years)	36.4 ± 1.5	36.4 ± 1.5	N/A	35.8 ± 6.8	35.8 ± 6.8	N/A	36.4 ± 7.7	36.4 ± 7.7	N/A	37.6 ± 4.1
Sex^†^ (female)	34 (68.0)	34 (68.0)	N/A	32 (47.0)	32 (47.0)	N/A	29 (69.0)	29 (69.0)	N/A	65 (64.7)
BMI^*^(kg/m^2^)	21.5 ± 2.5	21.4 ± 2.7	0.512	21.7 ± 2.2	21.6 ± 3.1	0.625	21.4 ± 2.3	21.1 ± 3.2	0.416	21.7 ± 3.0
Anterior knee pain^†^	35 (70.0)	25 (50.0)	0.087	17 (25.0)	27 (39.7)	0.121	23 (54.8)	28 (66.7)	0.180	90 (77.6)
Patellar tenderness (superior pole)^†^	5 (10.0)	7 (14.0)	0.754	4 (5.9)	6 (8.8)	0.754	27 (64.3)	14 (33.3)	**0.019**	45 (38.8)
Patellar tenderness (inferior pole)^†^	38 (76.0)	20 (40.0)	**0.001**	4 (5.9)	8 (11.8)	0.289	5(11.9)	9(21.4)	0.388	85 (73.3)

^*^Data: Mean ± SD. ^†^Data: No. (percentage)

p^a^ Comparison between SHFPE group cases and controls

p^b^ Comparison between QFPE group cases and controls

p^c^ Comparison between PFPE group cases and controls

Mixed group cases^d^: 124 cases with two or three different fat pad edemas

Abbreviations: BMI, Body mass index, SHFPE, superolateral Hoffa fat pad edema, QFPE, quadriceps fat pad edema; PFPE, prefemoral fat pad edema; SD, standard deviation

**Table 2:** Intra-observer and interobserver reliabilities in continuous variables

Variable	Intra-observer				Interobserver			
	ICC	Lower	Upper	p	ICC	Lower	Upper	p
TT-TG distance	0.959	0.919	0.979	**< 0.001**	0.944	0.891	0.972	**< 0.001**
TT-TG distance index	0.885	0.783	0.941	**< 0.001**	0.796	0.630	0.893	**< 0.001**
Trochlear depth	0.667	0.428	0.818	**< 0.001**	0.520	0.225	0.727	**0.001**
Lateral trochlear inclination	0.608	0.344	0.783	**< 0.001**	0.559	0.276	0.752	**< 0.001**
ISR	0.978	0.956	0.989	**< 0.001**	0.955	0.912	0.977	**< 0.001**
MISR	0.983	0.966	0.991	**< 0.001**	0.966	0.933	0.983	**< 0.001**
PTI	0.893	0.796	0.945	**< 0.001**	0.885	0.782	0.941	**< 0.001**
Patellar tilt angle	0.647	0.400	0.807	**< 0.001**	0.617	0.357	0.789	**< 0.001**
Bisect offset (%)	0.913	0.833	0.955	**< 0.001**	0.859	0.737	0.927	**< 0.001**

Abbreviations: TT, tibial tubercle; TG, trochlear groove; ISR, Insall-Salvati ratio; MISR, modified Insall-Salvati ratio; PTI, patellotrochlear index

### SHFPE and patellofemoral maltracking parameters

The mean TT-TG distance, TT-TG index, trochlear depth, patellar tilt angle, and bisect offset were not significantly different between cases and controls. Patients had a lower lateral trochlear inclination (21.7°±5.3° vs. 24.9°±5.0°; p = 0.028) than controls. ISR (1.35 ± 0.17 vs. 1.10 ± 0.15; p < 0.001) and MISR (2.25 ± 0.34 vs. 1.92 ± 0.25; p = 0.021) were both higher in cases, whereas PTI (0.31 ± 0.12 vs. 0.54 ± 0.14; p < 0.001) was lower in cases (Table 3).

**Table 3:** Superolateral Hoffa fat pad edema and patellofemoral maltracking parameters

		Mean ± SD		p-value
		SHFPE	Controls	
Lateralization of tibial tuberosity	TT-TG distance	8.6 ± 2.6 mm	6.7 ± 3.9 mm	0.055
	TT-TG index	0.13 ± 0.04	0.14 ± 0.16	0.607
Trochlear dysplasia	Trochlear depth	6.4 ± 2.0 mm	6.7 ± 1.4 mm	0.588
	Lateral trochlear inclination	21.7°±5.3°	24.9°±5.0°	**0.028**
Patella alta	ISR	1.35 ± 0.17	1.10 ± 0.15	**< 0.001**
	MISR	2.25 ± 0.34	1.92 ± 0.25	**0.021**
	PTI	0.31 ± 0.12	0.54 ± 0.14	**< 0.001**
Lateral patellar tilt	Patellar tilt angle	8.2°±4.5°	6.7°±3.9°	0.216
	Bisect offset (%)	59.4 ± 9.7	57.0 ± 8.1	0.305

Abbreviations: SD, standard deviation; TT, tibial tubercle; TG, trochlear groove; ISR, Insall-Salvati ratio; MISR, modified Insall-Salvati ratio; PTI, patellotrochlear index

### QFPE and patellofemoral maltracking parameters

Patellofemoral maltracking parameters were not significantly different between cases and controls (Table 4).

**Table 4:** Quadriceps fat pad edema and patellofemoral maltracking parameters

		Mean ± SD		p-value
		QFPE	Controls	
Lateralization of tibial tuberosity	TT-TG distance	7.7 ± 2.8 mm	7.4 ± 4.1 mm	0.773
	TT-TG index	0.13 ± 0.10	0.12 ± 0.07	0.255
Trochlear dysplasia	Trochlear depth	7.1 ± 2.2 mm	6.6 ± 1.6 mm	0.277
	Lateral trochlear inclination	24.5°±5.5°	24.6°±4.4°	0.931
Patella alta	ISR	1.16 ± 0.20	1.09 ± 0.13	0.108
	MISR	2.10 ± 0.31	1.90 ± 0.21	0.161
	PTI	0.45 ± 0.13	0.50 ± 0.02	0.068
Lateral patellar tilt	Patellar tilt angle	6.5°±3.9°	6.2°±3.1°	0.629
	Bisect offset (%)	57.9 ± 9.1	55.4 ± 6.2	0.243

Abbreviations: SD, standard deviation; TT, tibial tubercle; TG, trochlear groove; ISR, Insall-Salvati ratio; MISR, modified Insall-Salvati ratio; PTI, patellotrochlear index

### PFPE and patellofemoral maltracking parameters

The mean TT-TG distance, TT-TG index, and trochlear depth were not significantly different between cases and controls, but lateral trochlear inclination was (22.8°±5.5° vs. 26.8°±4.8; p = 0.014). ISR (1.34 ± 0.16 vs. 1.10 ± 0.12; p < 0.001) and MISR (2.30 ± 0.35 vs. 1.98 ± 0.12; p = 0.004) were both higher in cases, whereas PTI (0.35 ± 0.15 vs. 0.57 ± 0.16; p = 0.001) was lower. The mean patellar tilt angle (7.7°±4.1° vs. 5.3°±3.0; p = 0.019) and mean bisect offset (60.2%±7.9% vs. 54.1%±4.8%; p = 0.005) were significantly different between cases and controls (Table 5).

**Table 5:** Prefemoral fat pad edema and patellofemoral maltracking parameters

		Mean ± SD		p-value
		PFPE	Controls	
Lateralization of tibial tuberosity	TT-TG distance	8.1 ± 2.7 mm	7.6 ± 4.2 mm	0.678
	TT-TG index	0.13 ± 0.05	0.12 ± 0.07	0.496
Trochlear dysplasia	Trochlear depth	6.4 ± 1.3 mm	7.3 ± 1.9 mm	0.117
	Lateral trochlear inclination	22.8°±5.5°	26.8°±4.8°	**0.014**
Patella alta	ISR	1.34 ± 0.16	1.10 ± 0.12	**< 0.001**
	MISR	2.30 ± 0.35	1.98 ± 0.12	**0.004**
	PTI	0.35 ± 0.15	0.57 ± 0.16	**0.001**
Lateral patellar tilt	Patellar tilt angle	7.7°±4.1°	5.3°±3.0°	**0.019**
	Bisect offset (%)	60.2 ± 7.9	54.1 ± 4.8	**0.005**

Abbreviations: SD, standard deviation; TT, tibial tubercle; TG, trochlear groove; ISR, Insall-Salvati ratio; MISR, modified Insall-Salvati ratio; PTI, patellotrochlear index

### Association between SHFPE and other knee fat pads

There were 284 cases of peripatellar fat pad edema. Of the SHFPE cases, 134 were grade 0, 88 were grade 1, 17 were grade 2, and 45 were grade 3. Of the QFPE cases, 152 were grade 0, 80 were grade 1, and 52 were grade 2. Of the PFPE cases, 145 were grade 0, 81 were grade 1, and 58 were grade 2. SHFPE was negatively correlated (p < 0.001, r=-0.500) with QFPE and positively correlated (p < 0.001, r = 0.408) with PFPE (Table 6).

**Table 6:** Spearman correlation between superolateral Hoffa fat pad edema and other knee fat pad edemas

		QFPE	PFPE
SHFPE	r	-0.500	0.408
	p-value	**< 0.001**	**< 0.001**

Abbreviations: SHFPE, superolateral Hoffa fat pad edema; QFPE, quadriceps fat pad edema; PFPE, prefemoral fat pad edema

## Discussion

### Lateralization of the tibial tubercle

The TT-TG distance is a standard method for assessing lateralization of the tibial tubercle, and the TT-TG index was recently proposed [20] to account for knee size based on the TT-TG distance.

We found no difference or association for TT-TG distance or TT-TG index between the cases and controls in the SHFPE, QFPE, and PFPE groups, which was partly consistent with results of Subhawong et al. [10], who suggested no correlation between TT-TG distance abnormality and SHFPE, but contradicts with those of Kim et al. [12], who confirmed the association. This can be partly explained by the differences in determining the deepest point of the trochlear groove, particularly in the dysplastic trochlea. Furthermore, few patients in this study had a TT-TG distance greater than 15 mm, making direct comparisons with other studies difficult.

### Trochlear morphology

No subject had a trochlear depth below the 3-mm threshold for trochlear dysplasia, possibly due to low abnormal trochlear depth incidence. In our study, the lateral trochlear inclination was significantly lower in SHFPE and PFPE patients, meaning that a lower lateral trochlear inclination might lead to patellar maltracking and impinged IFP or PFP. Current studies [7, 11, 12, 27] have debated the relationship between SHFPE and trochlear morphology; however, most of them [12] did not mention if cartilage surface was included when measuring trochlear morphology, and they used different measurement planes such as 3 cm above the joint [11] or the most posterior condyle plane [7]. According to our experience, the trochlear morphology measurement should be along the osseous surface rather than the cartilage surface to decrease the effect of cartilage lesions, and the most posterior condyle plane was more convenient to measure and compare to the plane 3 cm above the joint. It seems that the effect of trochlear morphology on fat pad edema is limited compared with that of the patellar parameters.

### Patellar parameters

Abnormal patellar positions, such as patellar alta (high-riding patella), can lead to patellar maltracking [28]. Patellar alta has been measured on sagittal MRI sequences using different methods, including the ISR, MISR, and PTI. The most common method is the ISR since it is simple to measure and unaffected by knee flexion; however, it can be influenced by patellar morphology [29]. The MISR eliminates this disadvantage by measuring only a particular cartilage length and is more reproducible than the ISR; however, it can be affected by varied subchondral bone geometry [16]. The PTI truly represents patella-trochlear articulation since it quantifies the patella-trochlear contraction. Nevertheless, the PTI can be influenced by knee flexion [30]. A recent study [16] cautioned against using the ISR alone in clinical practice because of its poor agreement with other indices. Previous studies [16, 31] used cut-off values of 1.3 for ISR, 2.4 for MISR, and 0.18 for PTI on MRI. In our study, the mean ISR in SHFPE and PFPE was greater than 1.3, indicating that SHFPE and PFPE had more patella alta than controls. Although the mean MISR in SHFPE and PFPE did not reach 2.4, it was significantly greater than controls. The mean PTI in SHFPE and PFPE was more than 0.18, although significantly lower than controls. Patellar alta, a significant risk factor, is linked to SHFPE and PFPE, according to our findings.

Although the bisect offset between SHFPE and controls did not reach statistical significance, the PFPE groups had greater mean bisects than controls, implying that the effect of patellar maltracking on PFP might be greater than IFP. Such an association was found in PFPE with increased patellar tilt compared with the control. The subjects in our study were young and middle-aged patients without any dislocation history, implying that some patients prone to patellar dislocation who had abnormal patellar tilt might not have been included. Although Cilengir et al. [32] found a relationship between patellar tilt and peripatellar fat pad edema, they only included cases with patellar tilt greater than 5°, which differed from our study. According to our study, patellar abnormalities may be more important in causing SHFPE or PFPE than trochlear morphology abnormalities or lateralization of the tibial tubercle.

### Association between SHFPE and other knee fat pads

SHFPE and PFPE were more common in females according to our results, which might be related to the facts that females have a higher frequency of valgus knee and a larger Q angle than males [12]. These mechanical alignments may have a role in the development of SHFPE and PFPE in female patients to cause anterior knee pain. Superior or inferior poles of patellar tenderness may aid in the recognition of SHPFE and PFPE, however, differentiating them from patellar tendinopathy or quadriceps tendinosisis is still difficult due to the similar location. Therefore, MRI examination is necessary. Most studies [12, 27] focus on SHFPE and suggest a close association between SHFPE and patellar maltracking. However, there are few studies [33] on QFPE and PFPE, especially PFPE. Subhawong et al. [10] reported that PFPE was present in 68% of patients with knee pain and SHFPE. We found that PFPE was similar to SHFPE in that both were related to several maltracking parameters, particularly patellar alta, implying that both had a similar mechanical origin. We also found that SHFPE was positively correlated with PFPE and negatively correlated with QFPE. To our knowledge, no such association among these fat pad edemas has been reported, and we are the first to do so. The QFP and PFP are commonly thought to improve suprapatellar congruency of the extensor mechanism [1]. With the cushioning effect of the PFP [34], the patella does not contact the distal shaft of the femur when the knee is extended. When the knee is flexed, the patellar side moves downwards, while the trochlear side moves upwards to approach the patellar side and stabilize the knee joint. An opposite movement was found [35] to “sandwich” the PFP on the surface of the distal femoral cortex. Patellar maltracking might damage the PFP, leading to edema, which presents with an increased signal on PDW, similar to SHFPE [36]. However, according to Cosentino et al. [33], QFPE is unrelated to most imaging, clinical, and activity indicators. It is important not to overestimate their pathogenic significance, which is consistent with our findings. More histological and pathological studies are required to validate this. For clinical management, SHFPE and PFPE were different to QFPE according to our experience; treatment for QFPE might be unnecessary, but conservative treatment for SHPFE and PFPE should be recommended in patients with anterior knee pain. Conservative treatment includes [35, 37] activity modification, analgesics and non-steroidal anti-inflammatory drugs (NSAIDs), and physiotherapy. Physiotherapy for PFPE focuses on stretching the quadriceps and flexor muscles to help reduce the downward pressure of the patella on the PFP, which is different from SHFPE taping the patella in an upward position. IFP has been associated with osteoarthritis development and may predict knee osteoarthritis and knee replacement earlier [4, 38–40]. According to a nested case-control study by Li et al. [34], QFPE and PFPE are also linked to osteoarthritis. Peripatellar fat pads appear to have gained scholarly attention for their potential value.

### Limitations

There were several limitations in our study. Our measurements were based on static MRI, and we could not confirm whether the peripatellar fat pads impinged on dynamic activities. Due to the retrospective study design, the control group population consisted of subjects with mild abnormal pathology on MRI (such as mild tendinous, cartilaginous, or meniscal degeneration) rather than completely normal subjects, and it was impossible to compare activities of daily living between subjects, which could have affected the results. To mitigate this effect, we assigned age- and sex-matched controls for each case, and all the subjects were non-manual workers. We tried our best to reduce grading error and bias during grading of the fat pad scoring, but compared with morphological numerical measurements, grading is subjective. Although we graded the fat pad edema, it is difficult to perform pathological examinations in these cases, and correlated pathological changes were unknown. Finally, the sample size was small; additional research with larger sample sizes is required.

## Conclusions

In conclusion, SHFPE and PFPE were associated with patellar alta and lateral trochlear inclination. No association was observed between QFPE and patellofemoral maltracking parameters. SHFPE was positively correlated with PFPE and negatively correlated with QFPE, which is described for the first time in this study.

## Data Availability

The datasets used and/or analyzed during the current study are available from the corresponding author on reasonable request.

## Abbreviations

IFP: Infrapatellar (or Hoffa) fat pad
ISR: Insall-Salvati ratio
MISR: Modified ISR
MRI: Magnetic resonance imaging
NSAID: Non-steroidal anti-inflammatory drug
PDW: Proton density weight
PFP: Prefemoral fat pad
PFPE: Prefemoral fat pad edema
PTI: Patellotrochlear index
QFP: Quadriceps fat pad
QFPE: Quadriceps fat pad edema
SHFPE: Superolateral Hoffa fat pad edema
T1W: T1-weighted
TG: Trochlear groove
TT: Tibial tuberosity
